# Snow Avalanches and the Impact of Climate‐Linked Extreme Events on Mountain Wildlife Population Dynamics and Resilience

**DOI:** 10.1111/gcb.70415

**Published:** 2025-09-24

**Authors:** Kevin S. White, Taal Levi, Eran Hood, Chris T. Darimont

**Affiliations:** ^1^ Program on the Environment, Department of Natural Sciences University of Alaska Southeast Juneau Alaska USA; ^2^ Department of Geography University of Victoria Victoria British Columbia Canada; ^3^ Division of Wildlife Conservation Alaska Department of Fish and Game Juneau Alaska USA; ^4^ Department of Fisheries, Wildlife and Conservation Sciences Oregon State University Corvallis Oregon USA

**Keywords:** Alaska, avalanche, climate change, extreme events, mountain goat, *Oreamnos americanus*, population modeling, snow

## Abstract

Climate is changing rapidly in mountain environments, giving rise to increasing variability in weather, incidence of extreme events, and alteration of the cryosphere. Natural hazards, such as snow avalanches, and the ecological communities they impact may be particularly sensitive to such change. While avalanches may impose both ‘good’ and ‘bad’ effects on mountain ecosystems, the direct impacts that lead to mortality have particularly important implications for future viability and resilience of slow‐growing alpine wildlife populations. Here, we studied a sentinel species of coastal Alaskan mountain environments—the mountain goat ( *Oreamnos americanus* ) – using long‐term field data from individually marked animals (600 individuals over 44 years) in a quantitative modeling framework to understand how avalanches influence demographic processes. Specifically, we developed and parameterized a sex‐ and age‐specific population modeling approach to simulate the effects of avalanche‐caused mortality on population growth rate (*λ*). We examined a range of ecologically relevant scenarios based on empirically observed states of avalanche‐caused mortality. During years when avalanche impacts are severe, populations can experience significant additive mortality and population declines (up to 15%). Due to low reproductive rates, such impacts can lead to long demographic recovery times (up to 11 years, or ~1.5 mountain goat generations). Thus, during the course of a typical mountain goat lifetime, significant avalanche‐linked perturbations can be expected to occur, suggesting that meaningful demographic signatures of avalanche impacts are generationally recurrent and routinely imbedded in population histories. From a conservation perspective, such impacts are striking and highlight the utility of employing a quantitative modeling approach to predict possible effects of avalanches and extreme events more broadly on mountain ungulate population dynamics and viability. Our work explicitly builds upon recent findings about the importance of avalanches on mountain‐adapted animal populations and has implications for the cultural and ecological communities that depend on them.

## Graphical abstract

Climate is changing rapidly in mountain environments, increasing the incidence of extreme events and alteration of the cryosphere. Snow avalanches comprise a major source of mortality among slow‐growing mountain wildlife and have important implications for future viability and resilience. Long‐term data was used to develop a modeling framework to understand how avalanches influence demographic processes of mountain goats in coastal Alaska. Avalanches can elicit significant population declines, leading to long demographic recovery times (up to 1.5 generations). Such impacts are striking and highlight the potential role of avalanches, and extreme events more broadly, on mountain ungulate population dynamics and viability.

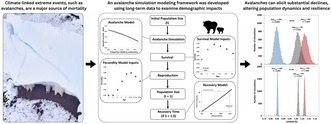

## 1 Introduction

Environmental change plays an important role in driving population dynamics and patterns of biodiversity (Ozgul et al. 2010; Bellard et al. 2012; Mills 2013; Gilbert et al. 2020). Increasingly, such dynamics are influenced by variation in weather conditions and linked to longer‐term processes of climate change (Bellard et al. 2012; Harris et al. 2018). Of principal concern is the increasing occurrence of extreme events that may have catastrophic, long‐lasting effects on populations and ecological systems (Sergio et al. 2018; Smith 2011b; Ummenhofer and Meehl 2017; Walsh et al. 2020). Such impacts may shift ecological baselines and hold the potential to compromise viability and even extirpate species, particularly specialists with narrow biophysical niches (Smith 2011a; Urban 2024). Despite such extraordinary impacts, our understanding of how extreme events affect populations is limited by the requirement to monitor populations for long periods of time to allow for the impact of rare events to be accurately quantified (Maxwell et al. 2019). Whereas important theoretical and empirical progress has been made, knowledge gaps remain, especially in sensitive, often difficult to study, ecological systems prone to severe weather and climatic variability (Smith 2011b; Ummenhofer and Meehl 2017).

Alpine ecosystems deserve special attention. Mountain environments comprise 25% of Earth's terrestrial surface, harbor a disproportionate fraction of global biodiversity, and are particularly sensitive to shifts in atmospheric conditions, including extreme events (Hock et al. 2019; Rahbek et al. 2019; Pepin et al. 2022; Urban 2024). Existing evidence indicates that climate is changing rapidly in mountain areas, altering snow and temperature regimes in significant ways and catalyzing impacts on sensitive ecological communities and processes (Pepin et al. 2022; Schmeller et al. 2022; Eckert et al. 2024). In these systems, the effects of climate change may be more strongly influenced by increases in the frequency of extreme events than by changes to average conditions. Snow cover in mountain regions, for example, is sensitive to shifts in weather and climate patterns due to changes in water vapor advection and air temperature lapse rates (Gultepe 2015). Specifically, due to orographic effects, mountains often receive substantial precipitation, with the fraction falling as rain or snow often hanging in the balance of small temperature fluctuations—but manifesting with outsized effects on the physical environment (Roe 2005; Shanley et al. 2015; Musselman et al. 2018). Such dynamics not only alter the abundance of snow but also its physical structure and stability.

**FIGURE 1:**
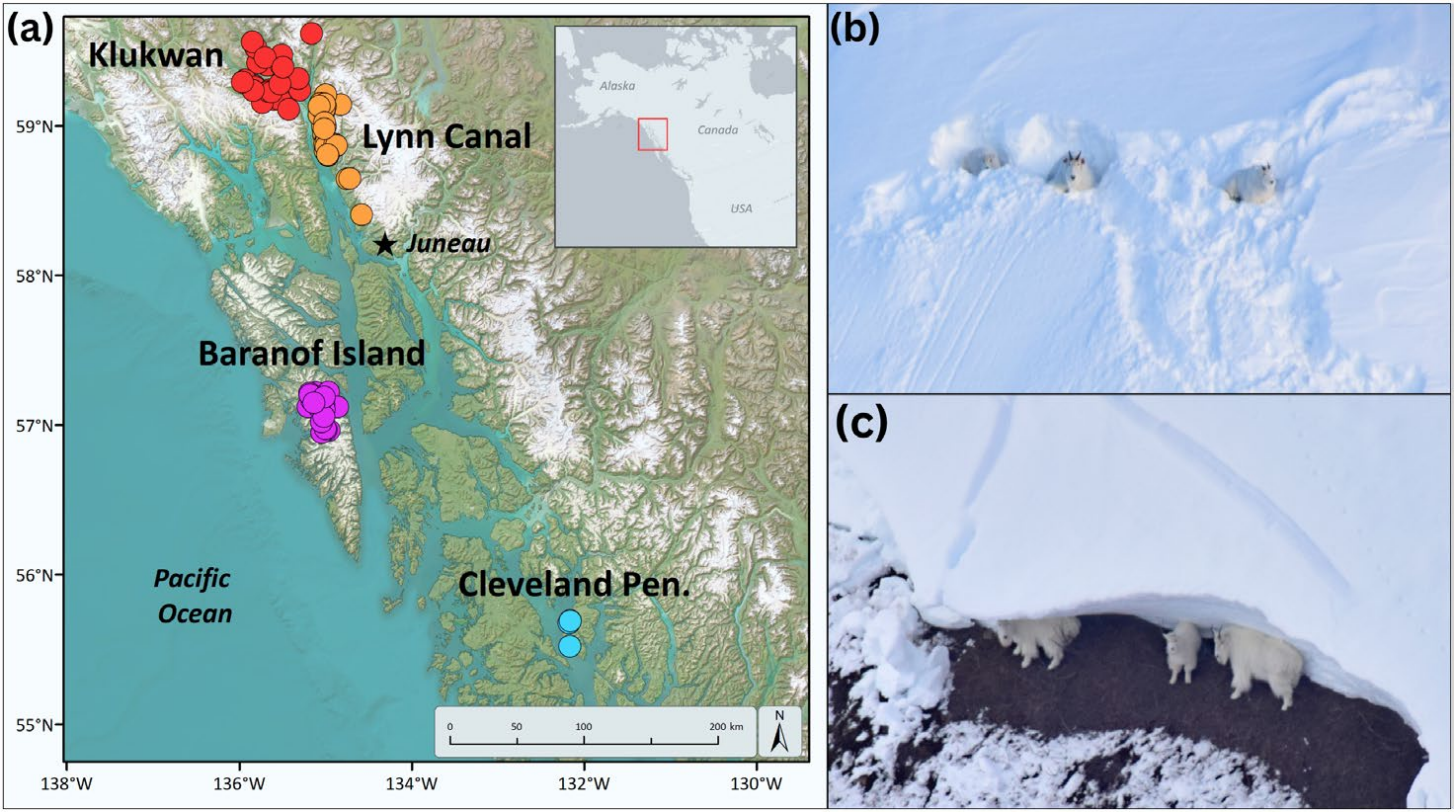
The mountain goat and avalanche study system. (a) Map depicting the four study areas and locations where radio‐marked mountain goats were studied and died in avalanches during 2005–2021 in coastal Alaska. (b) Mountain goats in extensively excavated beds within mapped avalanche terrain following an extreme snowfall event (2.4 m over 6 days) associated with an atmospheric river weather system during December 2020, near Porcupine Mountain, Klukwan, Alaska. (c) Mountain goats sheltering beneath the fracture line of a mid‐winter glide avalanche, Summit Creek, Klukwan, Alaska. (Photo credits: K. White).

The disproportionate impacts of climatic variability and extreme events in mountain environments carry important implications. Snow avalanches, for example, are driven by changes in the structure and stability of the snowpack and have been shown to exert strong ecological effects on diverse alpine plant and animal communities (Rixen et al. 2007; Bebi et al. 2022; White, Hood, et al. 2024). Specialized mountain wildlife populations can be particularly affected, with avalanches comprising a major source of mortality (Jonas et al. 2008; White, Hood, et al. 2024). For example, recent work indicated that annual mortality from avalanches can exceed 20% in some years for mountain goat ( *Oreamnos americanus* ; Figure 1) populations in coastal Alaska (White, Hood, et al. 2024). At such rates of mortality, avalanches could produce extreme demographic perturbations and represent an important climate‐linked driver of stochastic environmental change—that is, one that largely occurs at random and likely offers limited opportunity for local adaptation (Fisher et al. 2022; Hogarth et al. 2015; White, Hood, et al. 2024).

Mountain wildlife populations might be particularly vulnerable to stochastic and ecologically extreme events, given that animals in alpine ecosystems often persist in small, isolated populations. Bighorn sheep ( *Ovis canadensis* ), for instance, experience short‐term but intense and apparently unpredictable predation events that precipitate acute population declines, resulting in demographic restructuring and long recovery times (Festa‐Bianchet et al. 2006; Turgeon et al. 2024). Snow avalanches may have demographically analogous implications. Indeed, avalanches are an important agent of change in mountain systems and are difficult to predict, arising through multiple complex mechanistic pathways linked to terrain characteristics, synoptic meteorology, local weather chronology, snow pack evolution, and extreme events (McClung and Schaerer 2006; Schweizer et al. 2003). Yet, the impacts of snow avalanches may differ from predation events in important ways. For example, predation is selective and typically removes poorer quality young and old animals from a population; though some variation occurs depending on predator type (coursing versus ambush) and prey population composition (Festa‐Bianchet et al. 2006; Hoy et al. 2021; Rominger 2018; Turgeon et al. 2024). Conversely, avalanches largely remove animals at random, including a substantial fraction of reproductively critical, prime‐aged females (White, Hood, et al. 2024). Thus, the way stochastic extreme events impact population structure can vary, carrying important implications for population dynamics.

How populations might be affected by events such as avalanches will relate to life history characteristics. The severe environmental conditions in mountain ecosystems have led to specialized adaptations among alpine specialists, such as mountain goats, and typically involve conservative life‐history strategies, low growth rates, and resilience (Festa‐Bianchet and Côté 2008; Festa‐Bianchet et al. 2019). Individuals, for example, preferentially allocate energetic resources to survival at the cost of reproduction (*sensu* capital survivors; Stephenson et al. 2020). This adaptation has led to low reproductive and realized growth rates (1%–4%) among mountain goat populations (Hamel et al. 2006; Rice and Gay 2010; White, Levi, et al. 2021), relative to other northern ungulates. As a consequence, even small impacts can elicit deleterious changes that might entail prolonged recovery periods—raising pressing questions about the potentially important role stochastic processes and climate‐linked extreme events, such as avalanches, might play in population trajectories. To date, such dynamics have not been quantitatively examined, and the specific demographic implications of variability in avalanche‐linked mortality on population growth, viability, and recovery time are needed.

To advance our understanding of this important driver of mountain ungulate population dynamics and viability, we developed and implemented a population dynamics modeling approach for simulating the effects of varying levels of avalanche‐caused mortality on mountain goat population growth and recovery times. We parameterized a sex‐and age‐specific population projection model using a spatially and temporally extensive, 44‐year known‐fates data set collected from mountain goats throughout coastal Alaska (*n* = 14 study sites, 600 individuals) (White et al. 2011, 2018; White, Watts, and Beckmen 2021; White, Levi, et al. 2021, this study). Using a subset of the data for which avalanche‐caused mortality data were systematically recorded (*n* = 4 study sites, 421 individuals, 17 years; White, Hood, et al. 2024), we estimated the relationship between avalanche‐caused mortality and annual mountain goat survival. This sub‐model was used to quantify the degree to which avalanche mortality was additive vs. compensatory, and was integrated into our population model to simulate the effects of avalanche mortality on population growth rate (*λ*). We designed simulations to assess population responses across a range of scenarios encapsulating the variation observed during our long‐term avalanche mortality studies (*n* = 43 study area years) to explicitly examine the impacts of extreme avalanche‐caused mortality events. We also assessed the resilience of simulated populations to extreme events by calculating recovery time to baseline, pre‐avalanche levels (assuming populations resumed average growth rates following the simulated perturbation). We provided ecological context for our findings by estimating recurrence intervals of events in relation to key population biology criteria (i.e., generation time) of mountain goats. Overall, our modeling framework fills an important knowledge gap by providing realistic insights about avalanche impacts on mountain goat population dynamics to inform management and conservation strategies. More broadly, it provides a deeper understanding of the increasingly important role of climate‐linked perturbations and extreme events on population dynamics and resilience, particularly in mountain environments.

## 2 Methods

### 2.1 Study System

Mountain goats are a sentinel species of North American alpine ecosystems due to their sensitivity to variation in environmental conditions (White, Cadsand, et al. 2024). We studied mountain goats and avalanche relationships in four separate areas across a broad geographic range in coastal Alaska (5537 km^2^; Figure 1) from 2005 to 2021. This area is within the Coast Mountains biogeographic region (Gallant et al. 1995) and is largely characterized by maritime snow climate; though transitional snow climate characteristics occur in the northernmost part of the Klukwan study area (McClung and Schaerer 2006). Mean monthly temperatures range from −2°C to 14°C, and mean annual precipitation is 1400 mm in Juneau (Fellman et al. 2014), a regionally representative location. Across the region, annual precipitation ranges from 1 to > 8 m, and winter snowfall ranges from 0.5 to > 3 m of snow water equivalent (Shanley et al. 2015). During the study period, annual snowfall at sea level in Juneau averaged 233 cm, with a range of 89–501 cm (Juneau Forecast Office, National Weather Service, Juneau, AK).

The region is varied, rugged, and influenced by avalanche activity. It is dominated by coastal temperate rainforest, composed primarily of Sitka spruce‐western hemlock (*Picea sitchensis‐Tsuga heterophylla*) forests at lower elevations (below 450–750 m). At higher elevations, subalpine and alpine habitats dominated by krummholtz forest, low‐growing herbaceous meadows, and ericaceous heathlands are widespread and persist to elevations of about 1400 m. The geologic terrain is complex and strongly influenced by terrain accretion and uplift processes (Stowell 2006). The resulting landscape is highly fractured and dominated by steep, rugged topography that is fragmented by active glaciers, icefields, high‐volume river systems, and marine waters (Stowell 2006). The avalanche paths in this area extend from sea level to 2000 m and include a variety of aspects as a result of the complex topography of the Coast Mountains. Overall, 62% of the area used by mountain goats in this area is comprised of terrain delineated as avalanche hazard (White, Hood, et al. 2024).

Mountain goats in this region are widespread and occur at low to moderate densities (0.6–1.2/km^2^), typical of northern coastal areas inhabited by the species (White et al. 2016; White, unpublished data). Populations exhibit a high degree of local‐scale population genetic differentiation, with limited movement among geographically discrete mountain complexes (Shafer et al. 2012; White, Levi, et al. 2021). Mountain goats are habitat specialists and extensively utilize steep, rugged terrain in close proximity to cliffs—a behavioral strategy that mitigates the risk of predation but also increases vulnerability to avalanche mortality (Shafer et al. 2012; Sarmento and Berger 2020; White, Hood, et al. 2024). Mountain goats are partially migratory and exhibit seasonal variation in altitudinal distribution, with some individuals, depending on study area, residing in alpine and subalpine habitats throughout the year while others migrate to lower elevation wintering areas (Shakeri et al. 2021; White and Gregovich 2017, 2018). As such, wintering strategies are variable and influence their exposure to avalanche risk (White, Hood, et al. 2024).

### 2.2 Mountain Goat Monitoring

Male and female mountain goats (age range: 1–14 years) were captured using standard helicopter darting techniques (White, Watts, and Beckmen 2021). During handling, all animals were fitted with mortality‐sensing very high frequency (VHF) and/or global positioning system (GPS) radio‐collars (Telonics Inc., Mesa, AZ). Age of animals was determined by counting horn annuli and, in some cases, cross‐validated by examination of tooth eruption patterns (for young animals) (Smith 1988) and/or cementum analysis of incisors (for deceased animals; Matson's Laboratory, Milltown, MT). Following capture, animals were typically monitored at least once per month (often multiple times per month) via aerial telemetry to determine whether animals were alive or dead. Survival status was also determined via examination of GPS radio‐collar location, activity, and temperature sensor data, an approach that often enabled temporal determination of death to within a 6‐h time window. In cases where animals were determined to have died, an initial fixed‐wing aerial reconnaissance of the site was conducted and followed up with a ground‐based examination to determine context and causes of death, to the extent possible. Due to safety and logistic considerations, ground‐based examinations were typically conducted after initial aerial reconnaissance and determination of death. Due to the delay, it was not always possible to definitively distinguish among non‐avalanche‐related causes of death (i.e., due to scavenging of carcasses). However, avalanche‐caused mortality determinations were definitive and associated with carcasses being buried under, or associated with, avalanche debris and located within active avalanche paths. Capture and handling procedures complied with all relevant ethical regulations for animal use and were approved by the Alaska Department of Fish and Game Institutional Animal Care and Use Committee (protocols 05‐11, 2016‐25, 0078‐2018‐68, 0039‐2017‐39) and followed American Society of Mammalogists guidelines (Sikes and the Animal Care and Use Committee of the American Society of Mammalogists 2016).

### 2.3 Mountain Goat Population Modeling

**FIGURE 2:**
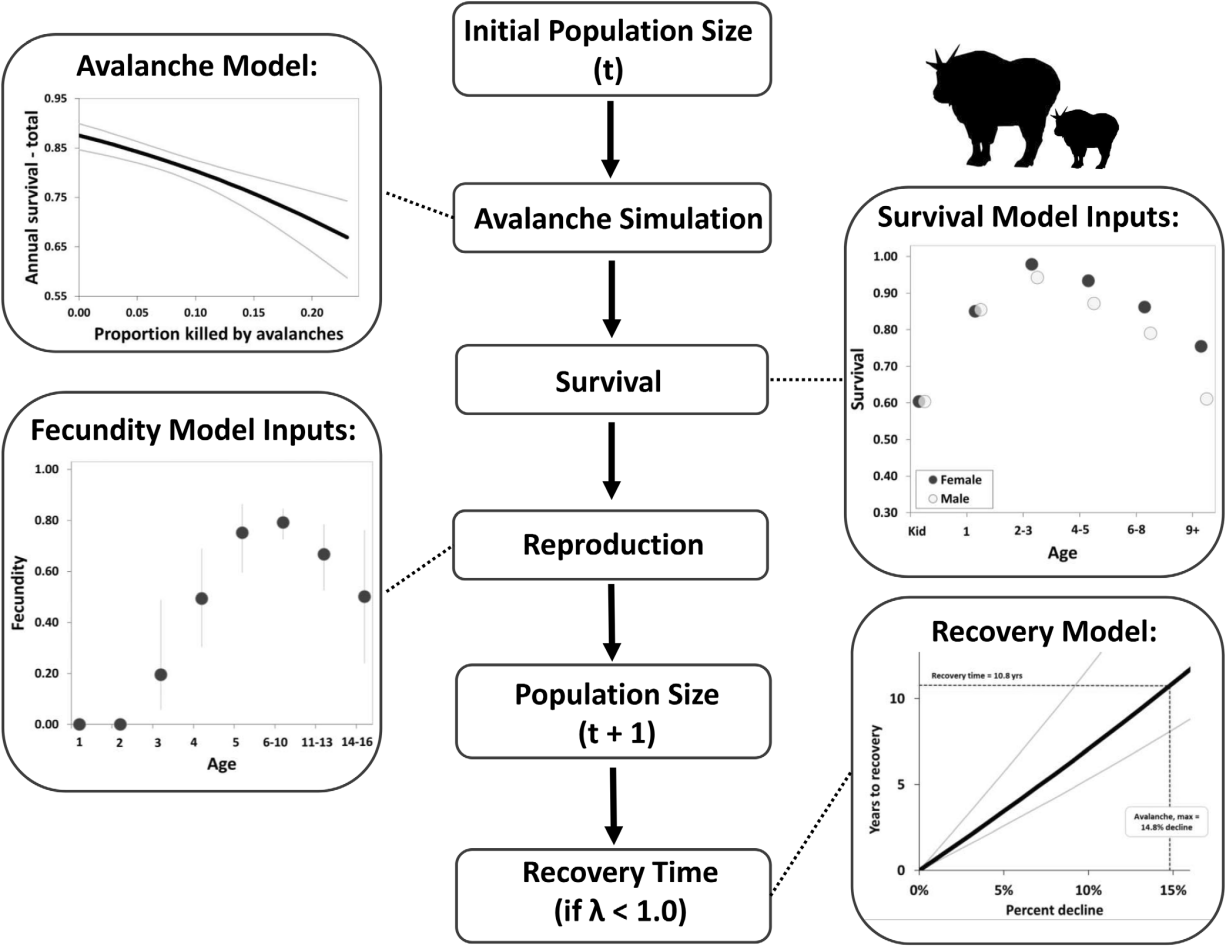
Conceptual diagram describing the dual‐sex, post‐breeding, age‐structured population model. The model accounts for variation in sex‐ and age‐specific fecundity and survival based on empirical data collected from radio‐marked individuals in coastal Alaska. The avalanche sub‐model (top left) modifies population size based on empirical relationships between avalanche mortality and annual survival, enabling scenario‐based simulation of avalanche impacts on population trajectories. Recovery time (bottom right) was calculated for scenarios resulting in population decline. Population growth during the recovery phase was modeled to coincide with average annual population growth observed during our study (*λ* = 1.015).

To examine demographic responses of mountain goat populations to simulated avalanche perturbations, we followed a multi‐step procedure (Figure 2). First, we updated and expanded a post‐breeding, sex‐ and age‐structured (20 age classes) population model previously described by White et al. (2018). Briefly, the model is parameterized using sex‐ and age‐specific vital rate estimates statistically derived using a spatially and temporally extensive, 44‐year (1977–2022) known‐fates data set collected from mountain goats throughout coastal Alaska [*n* = 14 study sites, 600 individuals (males = 295, females = 305), 1910 mountain goat years; Figures 2, S1, and S2] (White et al. 2011, 2018; White, Levi, et al. 2021, this study). Age‐specific fecundity was estimated based on direct observations of radio‐marked females (*n* = 180 females, 640 female‐years) during the parturition period in a subset of three study areas during a 16‐year period (2005–2021) (White et al. 2018; White, Levi, et al. 2021, unpub. data). Neonate survival was parameterized following Rice and Gay (2010), as described in White et al. (2018). Although the model was originally designed to simulate population trajectories given user‐specified climate inputs (White et al. 2018) and human harvest removals (White, Levi, et al. 2021), we simulated dynamics of non‐harvested populations at average climate conditions observed during the study. Such specifications were also used to calculate the stable stage distribution used for the initial population size input. To account for uncertainty, we sampled from within the error distribution of beta coefficients (i.e., sex‐ and age‐specific survival) accounting for covariance structure among coefficients using the RMark package in R (Laake 2013). We also modeled interannual variation in annual fecundity as a lognormally distributed random variable. The standard deviation of the distribution was parameterized using the observed variance across the range of interannual fecundity estimates (SD = 0.106, *n* = 16 years, 2006–2021; data described above). This approach enabled us to simulate demographic stochasticity using empirical data collected in our study system.

### 2.4 Modeling Effects of Avalanche Mortality on Survival

Using the framework described above to calculate annual change in population size over time, we next developed a sub‐model to simulate how avalanches remove individuals from a population across a range of different scenarios. The sub‐model was parameterized using a subset of our data for which cause‐specific mortality was systematically collected (*n* = 4 study areas, 421 individuals, 1218 mountain goat years, 2005–2021). While the complete (44‐year) data set used for developing the population model (see above) enabled derivation of the best possible estimates of sex‐ and age‐specific survival, it did not allow for estimation of avalanche‐specific mortality. Thus, we used a two‐step approach for simulating the effects of avalanches on population dynamics. First, we used the full data set to parameterize the population model; then, we employed the sub‐model to simulate avalanche impacts on projected population dynamics.

Avalanche risk is considered to be an unpredictable phenomenon, and mortality is expected to be largely additive and remove animals from a population at random (White, Hood, et al. 2024). To quantify the extent that avalanche mortality is additive and reduces overall survival, we estimated the relationship between the proportion of individuals within each life‐stage (following sex‐ and age‐structures defined by White et al. 2011, 2018) that died due to avalanches and total annual survival. Parameterization of this relationship enabled estimation of annual survival per life‐stage for any given rate of avalanche‐caused mortality. We estimated this relationship using a binomial generalized linear mixed modeling approach (package “lmer”, R version 4.3.1; Bates et al. 2014; R Core Team 2023). This model, parameterized using known‐fate data from each individual animal (coded based on whether an animal died due to avalanches, other causes or survived), was used to estimate annual survival for each life‐stage (following White et al. 2011) at the average population‐level avalanche mortality rate observed during our study (i.e., 7%; White, Cadsand, et al. 2024; White, Hood, et al. 2024). We also used the model to estimate annual survival, for each life‐stage, across the range of observed avalanche mortality values (i.e., 0%–23%; White, Cadsand, et al. 2024; White, Hood, et al. 2024). We then calculated the ratio between each of these survival values (i.e., specified at each avalanche mortality rate) and the survival value that occurred at mean avalanche mortality rate. This ratio, we refer to as the ‘proportional survival change factor’, was derived because estimated survival probabilities (used in the population model, see above) incorporate mortality due to all natural causes, including avalanches. Thus, the proportional survival change factor enabled adjustment of total annual survival in relation to changes in avalanche mortality from the mean. For example, annual survival at the user‐defined avalanche mortality is equal to the survival at average avalanche mortality multiplied by the change in survival in relation to varying avalanche mortality:

(1) $${Ŝ}_{\mathrm{avy}}={Ŝ}_{\mathrm{avy}\_\text{mean}}\times {Ŝ}_{\text{change}}$$

where, *Ŝ*_avy_ = annual survival at a specified level of avalanche mortality, *Ŝ*_avy_mean_ = annual survival at the mean level of avalanche mortality, and *Ŝ*_change_ = proportional survival change factor, or difference between survival estimated at the mean avalanche mortality value and the specified avalanche mortality value.

Thus, the proportional survival change factor represents the expected annual survival for a particular avalanche mortality rate divided by the survival at the mean avalanche mortality rate. For example, the proportional survival change factor is less than one if avalanche mortality increases above the mean value and greater than one if it declines toward zero. We computed the proportional survival change factor for every life‐stage, except neonates. As mentioned above, we parameterized offspring survival based on mean values reported in the literature (i.e., Rice and Gay 2010). However, to account for the effects of avalanche mortality on neonates, we used the slope derived from our generalized linear model and computed the intercept by setting annual survival to the reported neonate survival value (as described above) and avalanche mortality set to the mean avalanche mortality rate. This enabled derivation of a linear equation for predicting neonate survival on the logit scale with the same slope used for other life‐stages. This latter step involved an assumption that patterns of avalanche mortality among neonates were similar to older‐age classes; a reasonable assumption given the tight maternal bond (and spatial co‐occurrence) that persists through the first year of life between offspring and mothers and associated nursery groups (Festa‐Bianchet and Côté 2008).

### 2.5 Simulating Effects of Avalanche Mortality

To simulate avalanche impacts on mountain goat population dynamics, we first specified the population‐level avalanche mortality rate of interest and applied the appropriate proportional survival change factor for each life stage. During each year of the simulation, this resulted in a new population state vector at the current time step of the model to account for the change in survival when varying avalanche mortality. We then projected the population to the next time step (i.e., year) using matrix multiplication, which incorporates baseline life stage‐specific survival estimates derived using the entire long‐term data set (i.e., calculated at the mean avalanche mortality rate), as well as age‐specific fecundity estimates (described above).

To understand the implications of avalanches on mountain goat population growth and resilience, we conducted simulations across a range of avalanche mortality scenarios we observed during our long‐term studies (White, Cadsand, et al. 2024; White, Hood, et al. 2024). Specifically, we simulated avalanche mortality across a range of empirically derived percentiles (min = 0%, mean = 7%, max = 23%; Figure S3). We conducted 1000 simulations for each scenario over a specified time period (yearly increments) and summarized average annual population growth rate (*λ*). We chose an initial population size of 100 (comprised of all sex and age classes), which corresponds with common population sizes among mountain goats in coastal Alaska (test simulations using different initial population sizes resulted in qualitatively similar results). For each set of avalanche mortality scenarios, we conducted simulations across two different temporal extents. First, we conducted simulations over a 2‐year period. This simulation length was designed to emulate a single annual avalanche mortality event (i.e., the proportion of the population killed by avalanches over a single winter season). We also conducted simulations over a longer, 30‐year period (i.e., greater than 3 generations, IUCN 2012). In this case, the specified avalanche mortality rate was applied to each year of the simulation and was intended to emulate longer‐term avalanche mortality driven change in population growth. In practice, differences in estimated effects of avalanche mortality on average annual population growth rate were minimal with respect to the length (years) of simulation runs.

### 2.6 Recovery Time

In scenarios predicting population decline, we conducted a separate analysis to estimate the amount of time required for a population to re‐attain initial population size. For these calculations we assumed average population growth rate (i.e., *λ* = 1.015; see below) following the simulated avalanche event (population decline). Specifically, we derived the recovery time for a particular percent decline and population growth rate as follows. After a population decline is *x*%, the resulting population is $\left(1-x\right)N.$ For a population growing at rate lambda after time t this population will be $\left(1-x\right)N\lambda^{t}$. Setting this equal to the original population size N, we solve for the recovery time, t, to yield:

(2) $$\text{Recovery time}\;\left(\text{years}\right)=\frac{\log\left(\frac{1}{1-\%\text{decline}}\right)}{\log\left(\lambda \right)}$$

where, % decline represents the decline in the population under a given scenario (*x* above) and *λ* corresponds to the realized annual population growth rate under average conditions. To account for variability in our estimates of recovery time, we also derived estimates using the 25th and 75th percentiles of the average annual population growth distribution (i.e., *λ*, mean = 1.015, *λ*_25_ = 1.012, *λ*_75_ = 1.018). Ultimately, we calculated recovery time for three ecologically relevant scenarios anchored to the species demographic characteristics: (1) the maximum perturbation (population decline) observed (i.e., 23% of the population killed by avalanches), (2) the maximum perturbation expected to occur during a typical mountain goat lifetime (generation time = 7.2 years), and (3) the perturbation level that would require a generation for recovery. The frequency distribution of population‐level avalanche mortality events that occurred during our study (*n* = 43 study area years) was used to empirically estimate exceedance probability and ultimately recurrence interval (*sensu* Mays 2011) for each specified avalanche mortality scenario and associated recovery time calculation.

## 3 Results

### 3.1 Effects of Avalanche‐Caused Mortality on Total Annual Survival

**FIGURE 3:**
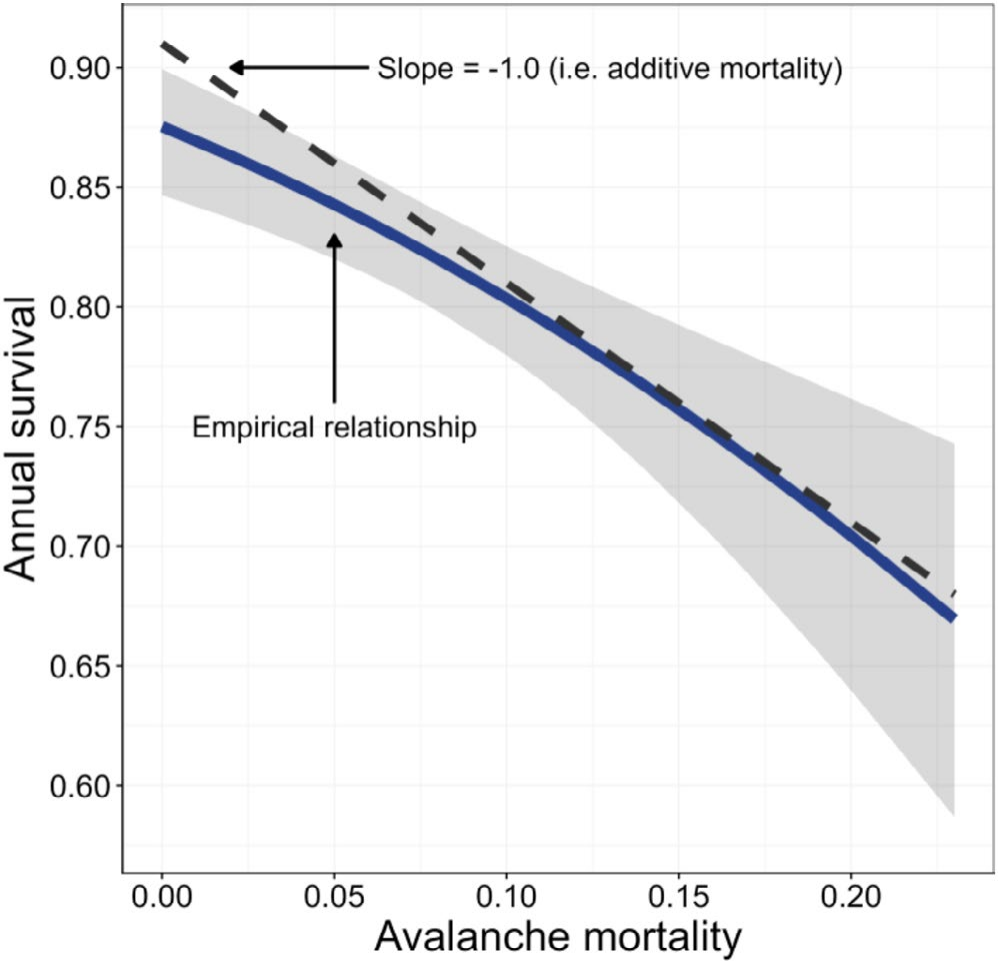
Diagram illustrating the relationship between the proportion of a mountain goat population dying in avalanches in a given year and total annual survival. The dashed black line illustrates a scenario where avalanche‐caused mortalities are completely additive. The solid dark blue line describes the empirical relationship based on radio‐marked mountain goats monitored in coastal Alaska during 2005–2021.

Generalized linear mixed effects modeling quantified the relationship between avalanche‐caused mortality and total annual survival of mountain goats, and was used to parameterize population modeling simulations (Table 1, Figures 3 and S4). This sub‐model was specified a priori to match the sex‐ and age‐structure used in the population model and earlier survival analyses (*sensu* White et al. 2011, 2018; White, Levi, et al. 2021). Annual survival estimates, under baseline (average) conditions, revealed similar sex‐ and age‐specific patterns, as compared to previous estimates in this study system (White et al. 2011). Relatively low survival was estimated for old males (*Ŝ* = 0.61 ± 0.07) and females (*Ŝ* = 0.77 ± 0.06) with prime‐age males (*Ŝ* = 0.82 ± 0.05) and females (*Ŝ* = 0.88 ± 0.04), young adults (*Ŝ* = 0.90 ± 0.04), subadults (*Ŝ* = 0.96 ± 0.04) and yearlings (*Ŝ* = 0.86 ± 0.19) exhibiting moderate to high annual survival. We specified the same generalized effect of avalanche mortality on annual survival across all sex‐ and age‐categories (sample size precluded rigorous examination of potential interactions; Table 1, Figures 3 and S4). Overall, this relationship revealed that avalanche‐caused mortality consistently tracked total annual survival when avalanche mortality was between mean and maximum levels, but decreased when avalanche‐caused mortality dropped below the mean (i.e., 7%; Figure 3). Thus, avalanche mortalities appear to be largely additive when occurring at high rates, while at low rates (< 7%) a small proportion of avalanche mortalities are compensatory.

**TABLE 1:** Generalized linear mixed effect model parameter estimates (*β*) and standard errors used to predict mountain goat annual survival in relation to proportion of the population killed by avalanches and life‐stage (sex‐ and age‐category) in coastal Alaska, 2005–2021. Individual identity was treated as a random effect, and the reference category was old males.

Parameter	*β*	SE	Confidence intervals		*Z*	*p*
			Lower	Upper		
Intercept	0.845	0.186	0.480	1.209	4.542	0.000
Avalanche mortality	−5.475	1.211	−7.849	−3.100	−4.520	0.000
Yearling	1.347	0.780	−0.182	2.876	1.727	0.084
Subadult	2.627	0.450	1.744	3.509	5.835	0.000
Young adult	1.742	0.254	1.245	2.239	6.869	0.000
Adult female	1.549	0.253	1.053	2.045	6.120	0.000
Adult male	1.048	0.218	0.619	1.476	4.797	0.000
Old female	0.741	0.230	0.291	1.191	3.230	0.001

### 3.2 Simulating Effects of Avalanche Mortality

**FIGURE 4:**
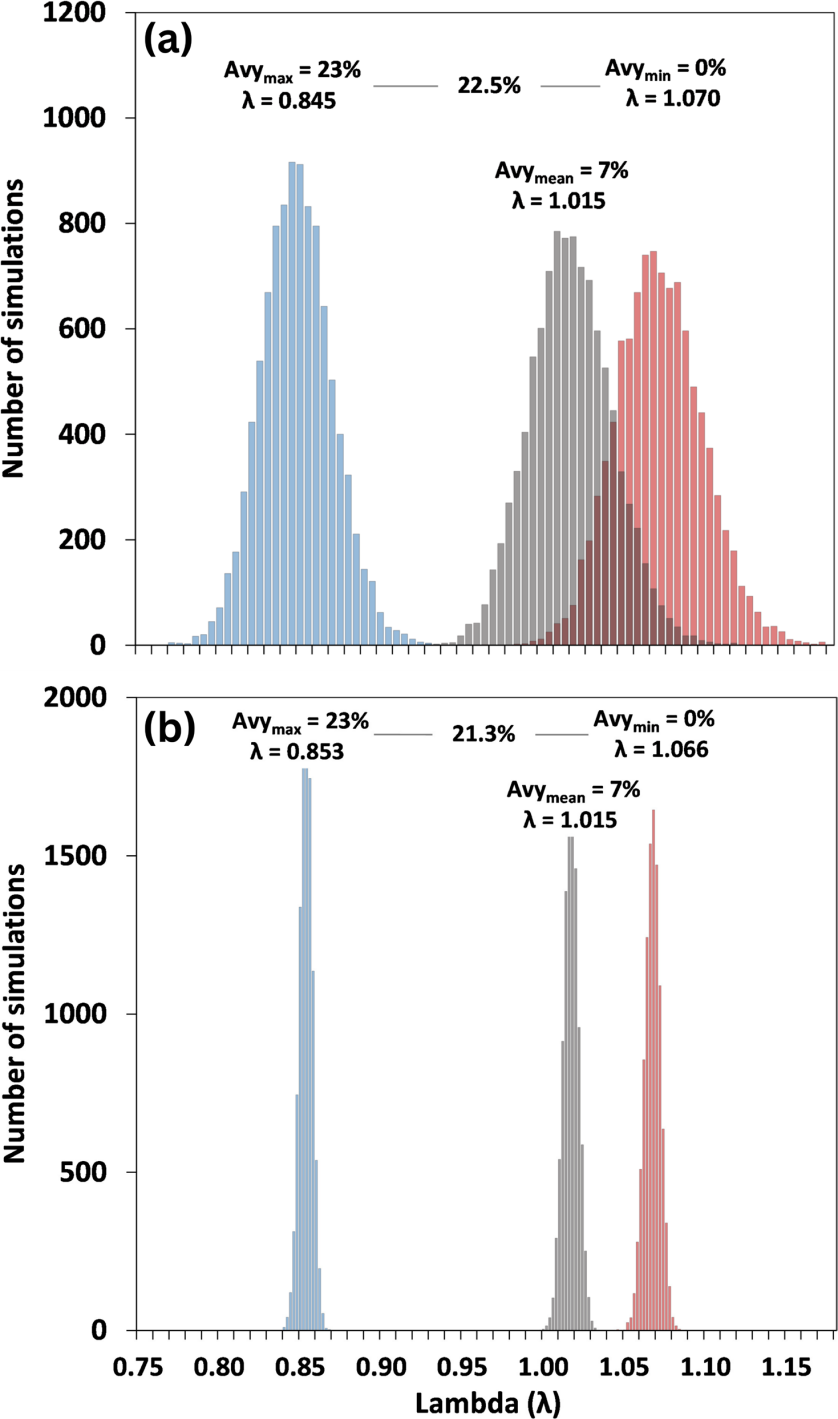
Variation in annual population growth (*λ*) across the range [minimum (Avy_min_), mean (Avy_mean_), and maximum (Avy_max_)] of avalanche‐caused mountain goat mortality observed in four areas of coastal Alaska during 2005–2021. Population growth estimates represent the average annual *λ* for each (a) 2‐year and (b) 30‐year model simulation. The difference in annual population growth between the ‘best case’ (Avy_min_) and ‘worst case’ (Avy_max_) scenario is annotated atop the panel. Each scenario was simulated 1000 times by randomly drawing input values from each parameter coefficient error distribution.

To illustrate how avalanche‐caused mortality can influence population growth rates across a range of scenarios, we incorporated sex‐ and age‐specific avalanche mortality relationships into our analytical framework and implemented population modeling simulations. Short‐term (2‐year) single winter avalanche simulations indicated that under average conditions (i.e., 7% of a population killed by avalanches) mountain goat populations were expected to exhibit relatively low annual rates of growth (*λ* = 1.015, 25th percentile *P*_25_ = 0.999, 75th percentile *P*_75_ = 1.033, Figure 4a). Under conditions where no animals were killed by avalanches, populations were expected to exhibit 7.0% annual population growth (*λ* = 1.070, *P*_25_ = 1.053, *P*_75_ = 1.089; Figure 4a). In contrast, during severe conditions (23% population‐level avalanche mortality), populations were estimated to decline 15.5% (*λ* = 0.845, *P*_25_ = 0.831, *P*_75_ = 0.860; Figure 4a). Thus, across the range of conditions observed, avalanche mortality is capable of shifting annual population growth by 22.5% (Figure 4a).

Longer‐term (30‐year) simulations revealed similar patterns of avalanche impacts on average annual population growth across scenarios but showed less variation within simulations (Figure 4). Under average avalanche mortality conditions, populations exhibited low annual growth rates (*λ* = 1.015, *P*_25_ = 1.012, *P*_75_ = 1.018, Figure 4b). In contrast, the average annual population growth rate was higher (*λ* = 1.066, *P*_25_ = 1.063, *P*_75_ = 1.070, Figure 4b) when no animals were killed by avalanches and substantially lower under the most severe conditions (*λ* = 0.853, *P*_25_ = 0.850, *P*_75_ = 0.855, Figure 4b). Across the range of scenarios examined, average annual population growth varied by 21.3% (Figure 4b). Notably, the 30‐year time period simulations enabled the determination of the change in average annual avalanche‐caused mortality required to cause populations to decline over the long‐term. Specifically, if average annual avalanche mortality shifted from the current baseline (7%) to 8.8%, populations were predicted to have an equal likelihood of increasing or decreasing. That is, at this threshold, ~50% of simulations predicted population decline over a 30‐year period.

**FIGURE 5:**
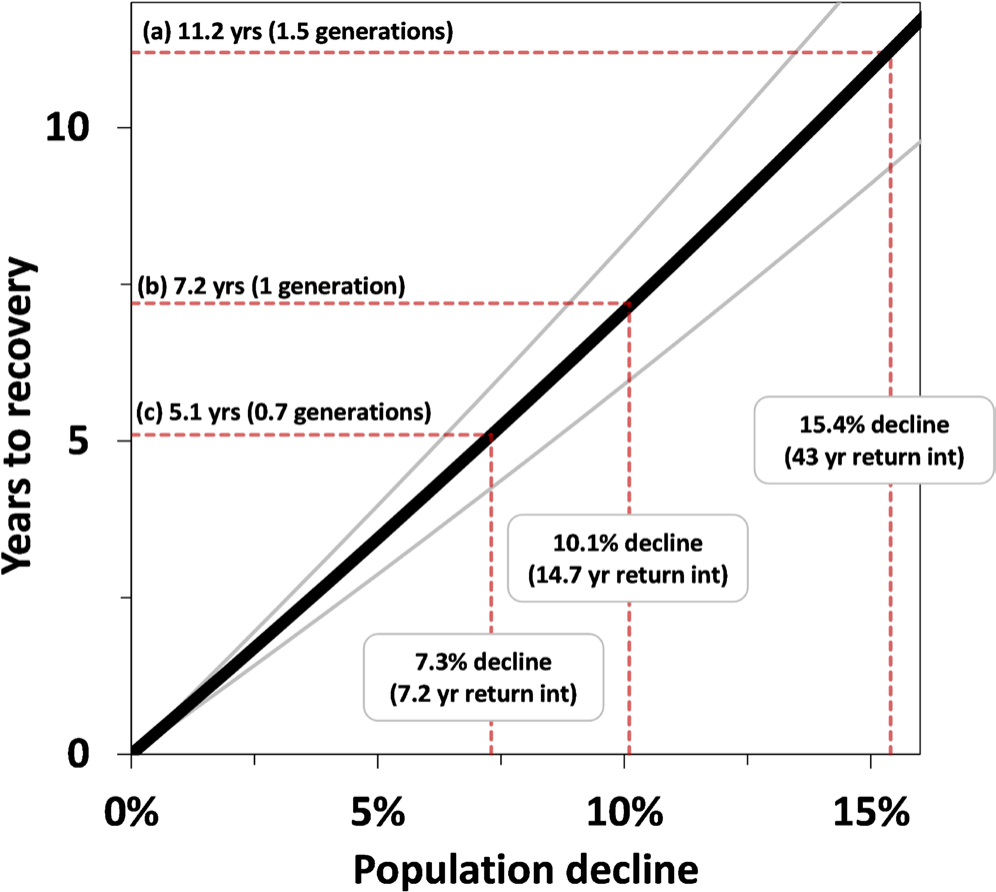
Mountain goat population recovery time estimated in relation to population declines associated with ecologically relevant avalanche impact scenarios. (a) worst‐case scenario, illustrating the maximum percent of a population observed killed by avalanches (23%), resulting in a 15.4% population decline and requiring 11.2 years (~1.5 mountain goat generations) to recover to original population size (at average population growth rates, *λ* = 1.015), (b) generational recovery scenario, illustrating a simulated perturbation (10.1% decline) requiring ~one mountain goat generation (7.2 years) for recovery; (c) once in a generation scenario, a simulated perturbation (7.3% decline) expected to occur during a typical mountain goat generation (every 7.2 years), and requiring 5.1 years for recovery.

We simulated population dynamics and recovery time associated with several illustrative scenarios that involved population declines. First, we considered the most extreme event (23% population‐level avalanche mortality, 15.5% population decline, 43 year recurrence interval, 2.3% annual chance of occurrence); followed by average annual conditions during the recovery phase. Under such a scenario, we determined that population recovery would not be attained for 11.2 years (*P*_25_ = 9.4, *P*_75_ = 12.9 years; Figure 5). We also calculated recovery time under a scenario of the maximum perturbation expected to occur during a typical mountain goat lifetime (16% population‐level avalanche mortality, 7.3% population decline, 7.2 year recurrence interval, 14% annual chance of occurrence) and determined that 5.1 years (0.7 mountain goat generations) would be required for recovery (Figure 5). Finally, we estimated it would take one mountain goat generation (7.2 years) for a population to recover following a year when 19% of the population was killed by avalanches (10.1% population decline, 14.3 year recurrence interval, 7% annual chance of occurrence; Figure 5).

## 4 Discussion

Avalanches represent a major climate‐linked driver of mountain goat populations and are capable of catalyzing extreme biological responses. Previous analyses revealed that avalanches comprise 36% (and up to 65%, depending on area) of mountain goat mortalities, and that prime‐aged, reproductively critical individuals are heavily impacted (61% of all mortalities) (White, Hood, et al. 2024). Translated to the population level, an average of 7% of a given population was estimated to be killed by avalanches annually, with mortality exceeding 22% in severe years (White, Hood, et al. 2024). Such high rates of mortality are striking and carry important implications for the viability and resilience of slow‐growing, mountain‐adapted wildlife populations. Our population modeling approach, parameterized using extensive, long‐term data, provided important quantitative insight and revealed that avalanches can elicit major population declines requiring extended periods (multi‐generational, in some cases) for recovery to baseline levels. That avalanches are capable of exerting such pronounced and long‐lasting impacts on populations has not previously been documented and represents an important, new advance in our understanding of the effects of climate‐linked factors and associated change on mountain wildlife populations.

Snow influences population dynamic processes in complex ways, often exerting strong impacts in mountain ecosystems. Ecological pathways through which snow influences demography have been extensively studied and include nutritional limitation (forage burial and nutritional dynamics), energetic costs of locomotion, and predation risk (Penczykowski et al. 2017; Boelman et al. 2019; Reinking et al. 2022). These processes typically result in selective, often compensatory, removal of young and old individuals that are predisposed to mortality due to heightened vulnerability, incomplete physical development, or otherwise poor body condition (Gilbert et al. 2020; LaSharr et al. 2023). Avalanches, on the other hand, represent a direct physical mechanism through which snow kills animals, largely removing individuals from a population at random, including reproductively critical prime‐aged individuals that otherwise exhibit high survival (White, Hood, et al. 2024). Our modeling work reveals important insights about these dynamics and involved empirical determination that avalanche mortality is primarily additive, except at low levels. That is, avalanches largely kill animals that would have otherwise survived (thus adding to baseline mortality rates), yet at low levels a small fraction of animals killed by avalanches would have died anyway (i.e., compensatory mortality). Under average conditions, individuals are expected to have adequate fat reserves sufficient for reproduction and survival, and buffered against the risk of malnutrition (Bårdsen et al. 2008; Parker et al. 2009). Translated into an applied example, our scenario‐based simulations illustrated that mountain goat populations experiencing average levels of avalanche mortality are capable of exhibiting relatively modest population growth (i.e., *λ* = 1.015, similar to what has been described elsewhere for the species; Hamel et al. 2006, Rice and Gay 2010; White, Watts, and Beckmen 2021; White, Levi, et al. 2021). However, during severe years, avalanche mortality is capable of eliciting significant population declines (~15%) that can require extended periods (~11 years, or 1.5 mountain goat generations) before populations recover to baseline levels, provided average conditions persist during the recovery phase.

In the context of the species' biology and life‐history characteristics, avalanches can clearly precipitate extreme demographic effects. Impacts may be exacerbated if conditions driving avalanche mortality, and associated demographic impacts, are temporally related to weather patterns that persist across consecutive years, leading to commensurately large cumulative demographic effects. For example, large‐scale atmosphere‐oceanic oscillations, such as the Oceanic Niño Index and other Pacific Oscillation indices, influence winter weather and avalanche occurrence and risk in mountain ecosystems across western North America (Mantua and Hare 2002; Haegeli et al. 2021), including within our coastal Alaska study area (Peitzsch et al. 2023). Alternating warm and cool phases associated with large‐scale atmosphere‐oceanic oscillations typically persist for multiple years (Mantua and Hare 2002) and hold the potential to impose multi‐year cumulative impacts on mountain goat demography via impacts to avalanche frequency. As such, our single‐year avalanche impact scenarios are likely conservative, and negative impacts can be greater than we illustrate when multi‐year climate oscillations occur. Alternatively, recovery times may accelerate if multiple persisting ‘good’ years follow a deleterious impact. These insights can have important practical implications. In an analytical context, for example, explicitly considering such dynamics can improve the realism of modeling projections and may be especially useful in applied settings where empirical field observations are available and allow for custom tuning of modeling simulations and projecting population performance.

The long‐term data used to parameterize our modeling approach offers important insights about current and, potentially, projected future population dynamics for mountain goats in coastal mountain systems. For example, our data revealed that over the course of typical generations, a population is expected to experience an avalanche‐caused mortality event eliciting a 6.8% decline, requiring 4.8 years for recovery to baseline levels. Thus, over the course of a mountain goat's lifetime, an individual is likely to experience a significant avalanche‐linked population perturbation, with associated transient demographic effects altering population structure, impacting specific cohorts, and exerting long‐lasting effects (*sensu* Gilbert et al. 2020; Turgeon et al. 2024). Climate‐linked impacts of avalanches are therefore meaningfully embedded in the history and trajectory of mountain goat populations on a routine, generationally recurrent basis. The impacts of these events, therefore, cannot be solely regarded as rare or extreme cases in isolation. Extreme events can result in severe (and long‐lasting) demographic responses and are of particular interest in relation to climate‐linked phenomena (Ummenhofer and Meehl 2017; Maxwell et al. 2019). Shifting climate baselines and associated distributional extremes suggest historically rare events are likely to be more common (Smith 2011b). While extreme events are often defined using statistical criteria (i.e., 5% of occurrences, or years), from an ecological perspective, it can be equally important to situate them in relation to a species' life‐history characteristics and capacity to recover from perturbations (Bailey and Van De Pol 2016).

Whereas mountain goat populations have the capacity to recover from snow avalanche impacts, the benefits of mild conditions appear to be outweighed by the negative impacts of severe events. For example, in the most deleterious avalanche‐caused mortality scenario, populations are expected to decline 17.0% (*λ* = 0.845) below baseline (*λ* = 1.015) levels, whereas during the most extreme positive scenario, populations were only expected to increase 5.5% above the baseline (*λ* = 1.070). Thus, the benefits of mild conditions are unable to equivalently compensate for the negative effects that occur during winters with severe conditions. Avalanches have particularly strong effects on population dynamics because they act upon the vital rate (survival) that exerts the strongest effect on the species' population growth (Gaillard et al. 2000; Hamel et al. 2006), with negative impacts spanning a broader range of possible outcomes than positive effects. Considered in relation to climate change forecasts—that project distributional shifts in snow and greater climatological variability—favorable conditions (years with low avalanche mortality) would need to occur 2–3 times more frequently than extreme negative events to compensate for impacts, all other conditions being equal. Only in the Lynn Canal population did we observe a low occurrence of years (3 of 16 years, 18.7%) in which avalanches were predicted to elicit population declines (Figure S5). In other populations, years with predicted avalanche‐driven declines were more common, occurring in about 1 of every 3 years on Baranof Island (4 of 11 years, 36.3%) and nearly as common as favorable years in the Klukwan population (5 of 11 years declining, 45.4%; Figure S5).

Whether the rate of avalanche occurrences documented here persists over longer time horizons in the face of projected changes in climate is uncertain. Avalanches already represent a hard to perceive risk for animals (i.e., a ‘wicked problem’; Hogarth et al. 2015; Fisher et al. 2022; White, Cadsand, et al. 2024; White, Hood, et al. 2024) and, according to recent syntheses (Eckert et al. 2024), patterns of avalanche risk are expected to change going into the future. Atmospheric rivers, for instance, and the extraordinary snowfalls they deposit in high elevation areas (Figure 1b), are expected to increase in frequency with climate change (Payne et al. 2020) and are disproportionately linked to elevated avalanche occurrence and risk (Hatchett et al. 2017). Such relationships represent an important mechanistic example of how extreme events can affect avalanche processes and, ultimately, the demography of alpine wildlife. Upward shifts in snow linked to warmer winter temperatures (Shanley et al. 2015), however, may exert countervailing impacts and illustrate an alternative pathway by which mountain wildlife might increasingly benefit from projected climate‐linked changes in snowcover and avalanche dynamics. Seasonal shifts in climate patterns, including increased variability, may also independently affect performance through ecological pathways (White, Cadsand, et al. 2024) and compound, or even offset, avalanche impacts. Despite such uncertainties, the future prevalence of ‘good’ vs ‘bad’ avalanche years, and their powerful demographic effects, will likely loom large over the fate of mountain goats and other similar mountain species.

Climatic variability represents an increasingly important driver of population dynamics and persistence worldwide (Parmesan 2006; Scheffers et al. 2016; Urban 2024). The pressing challenges associated with understanding and mitigating forecasted changes highlight the need to develop and implement analytical frameworks to acquire reliable and actionable knowledge. In this study, we describe and highlight the major role that ecologically extreme events, specifically avalanches, play in influencing the population dynamics and resilience of a culturally and ecologically significant mountain wildlife species. Integration of such knowledge into quantitative modeling approaches, such as those described here, represents a key tool for advancing mechanistic insights about the underlying demographic consequences of avalanches on population dynamics and for examining conservation‐relevant scenarios. For example, our modeling approach enables examination of how variation in climate‐linked processes explicitly impacts population size, composition, growth rate, and ultimately, recovery time. Such knowledge is critical for devising appropriate, time‐sensitive conservation decisions (seasonal management of land use and hunting) and for supporting longer‐term strategic planning (climate adaptation strategies, wildlife conservation plans). Application of this and similar demographic modeling approaches has traditionally been underutilized among studies at the climate–ecology interface (Urban 2024) but offers an important means for assessing the underlying mechanisms and impacts of environmental variation on demography, viability, and resilience of climate‐sensitive species. Such efforts are especially needed in mountain ecosystems that are otherwise relatively understudied yet particularly vulnerable to climate‐linked impacts and extirpations (Marris 2007; Schmeller et al. 2022; Urban 2024; White, Cadsand, et al. 2024).

If climate change alters the distribution, frequency, and character of avalanches, as has been suggested (Ballesteros‐Cánovas et al. 2018; Giacona et al. 2021; Eckert et al. 2024), possible futures may include restructuring of mountain communities. Globally, thirty‐two species of mountain ungulates across 70 countries occupy rugged, often avalanche‐prone, alpine terrain. Many of these species exhibit similarly low demographic capacity, with several species already considered vulnerable or imperiled (Shackleton 1997). That avalanches comprise a major pathway by which snow can elicit substantial demographic effects on slow‐growing, mountain‐adapted animal populations has only recently been reported (White, Cadsand, et al. 2024; White, Hood, et al. 2024). Our work here builds upon initial descriptions, quantifying in detail the pronounced, sometimes multi‐generational, impacts that avalanches can impose on mountain goat populations. The implications of such findings can be far‐reaching. Mountain goats are an iconic species of North American mountain landscapes and their viability is central to the persistence of cultural and subsistence relationships that have endured for millennia (Rofkar 2014; Jessen et al. 2022; Greening (La'goot) 2024). The species also plays a key role in nutritionally subsidizing mountain carnivore food webs that rely on mountain goats, including those killed in avalanches (Figure S6). Thus, understanding the extent that mountain goat populations fluctuate in response to avalanches offers deeper insight into the functionality and dynamics of mountain ecosystems and communities—relationships that are tightly coupled with future changes in the climate and the cryosphere.

## Author Contributions

**Kevin S. White:** conceptualization, data curation, formal analysis, funding acquisition, investigation, methodology, project administration, validation, visualization, writing – original draft, writing – review and editing. **Taal Levi:** formal analysis, investigation, methodology, validation, writing – review and editing. **Eran Hood:** funding acquisition, project administration, supervision, writing – review and editing. **Chris T. Darimont:** supervision, writing – review and editing.

## Conflicts of Interest

The authors declare no conflicts of interest.

## Supporting information


**Figure S1:** Map depicting mountain goat distribution in Alaska, USA and northwestern Canada.
**Figure S2:** Mountain goat life cycle graph describing the dual‐sex, post‐breeding, age‐structured population model (adapted from White et al. 2018).
**Figure S3:** Spatiotemporal variation among populations in avalanche‐caused mortality.
**Figure S4:** The relationship between the proportion of a sampled mountain goat population dying in avalanches in a given year and total annual survival.
**Figure S5:** The relationship between proportion of a mountain goat population killed by avalanches across time for four study areas in coastal Alaska during 2005–2021.
**Figure S6:** Carnivore scavenging of mountain goat carcasses.

## Data Availability

The data and code that support the findings of this study are openly available via Zenodo at https://doi.org/10.5281/zenodo.16431688. Mountain goat location data are administered by the Alaska Department of Fish and Game, Division of Wildlife Conservation, and are not freely available due to conservation concerns [Alaska Statute 16.05.815(d)] but may be requested by qualified parties through a data sharing agreement.
