# Supplementary material for: Snow Avalanches and the Impact of Climate‐Linked Extreme Events on Mountain Wildlife Population Dynamics and Resilience
Source: Glob Chang Biol. 2025 Sep 24;31(9):e70415. doi: 10.1111/gcb.70415 (PMC12457988; doi:10.1111/gcb.70415)

**Supplemental information for:**

**Snow avalanches and the impact of climate-linked extreme events on mountain wildlife population dynamics and resilience**

Kevin S. White^1,2,3,*^, Taal Levi^4^, Eran Hood^1^, and Chris T. Darimont^2^

**Affiliations:** ^1^Program on the Environment, Department of Natural Sciences, University of Alaska Southeast; Juneau, AK 99801, USA, ^2^Department of Geography, University of Victoria; Victoria, BC V8W 2Y2, Canada, ^3^Division of Wildlife Conservation, Alaska Department of Fish and Game; Juneau, AK 99811, USA (Ret.), ^4^Department of Fisheries, Wildlife and Conservation Sciences, Oregon State University; Corvallis, OR 97331, USA.

**Corresponding author:**

Kevin White, Program on the Environment, Department of Natural Sciences, University of Alaska Southeast; Juneau, AK 99801, USA.

email: [kwhite27@alaska.edu](mailto:kwhite27@alaska.edu); Orcid : 0000-0002-5231-6045

Figure S1. Map depicting mountain goat distribution in Alaska, USA and northwestern Canada. Study sites (red circles) where field data were collected to parameterize mountain goat survival models for input into the dual-sex, post-breeding, age-structured population model (n = 14 study sites, 600 individuals, 1,910 mountain goat yrs, 1977 – 2022; White et al., 2011, 2018, this study). Juneau, Alaska is depicted by the white triangle for reference. Adapted from White et al. 2018.


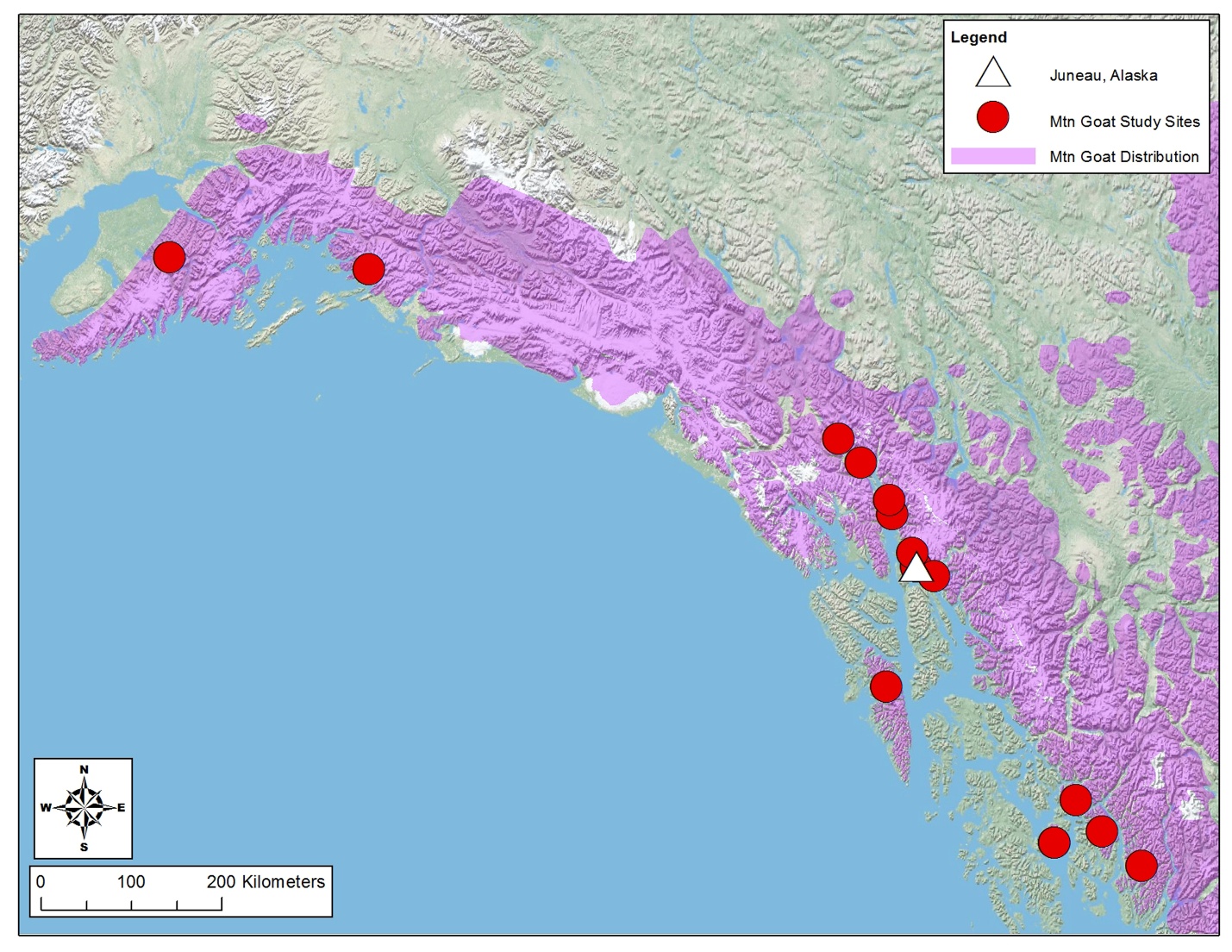


Figure S2. Mountain goat life cycle graph describing the dual-sex, post-breeding, age-structured population model (adapted from White et al. 2018). The model is implemented with 20 age classes (kids to 19+) for each sex but is simplified as a stage-structured model in this life cycle diagram for display purposes. Model description: S_0_ = kid survival (no sex effect; based on values from Rice & Gay, 2010), S_1_ = yearling survival (White et al., 2011, 2018, this study), S_2,3_ = 2- and 3-year-old survival (White et al., 2011, 2018, this study); S_4,5_ = 4- and 5-year-old survival (White et al., 2011, 2018, this study), Sm_6,7,8_/Sf_6,7,8_ = 6-, 7-, and 8-year-old survival (White et al., 2011, 2018, this study); Sm_9+_/Sf_9+_ = 9+-year-old survival (White et al., 2011, 2018, this study); r_3–12+_ = age-specific fecundity (White et al. 2018, unpublished data).


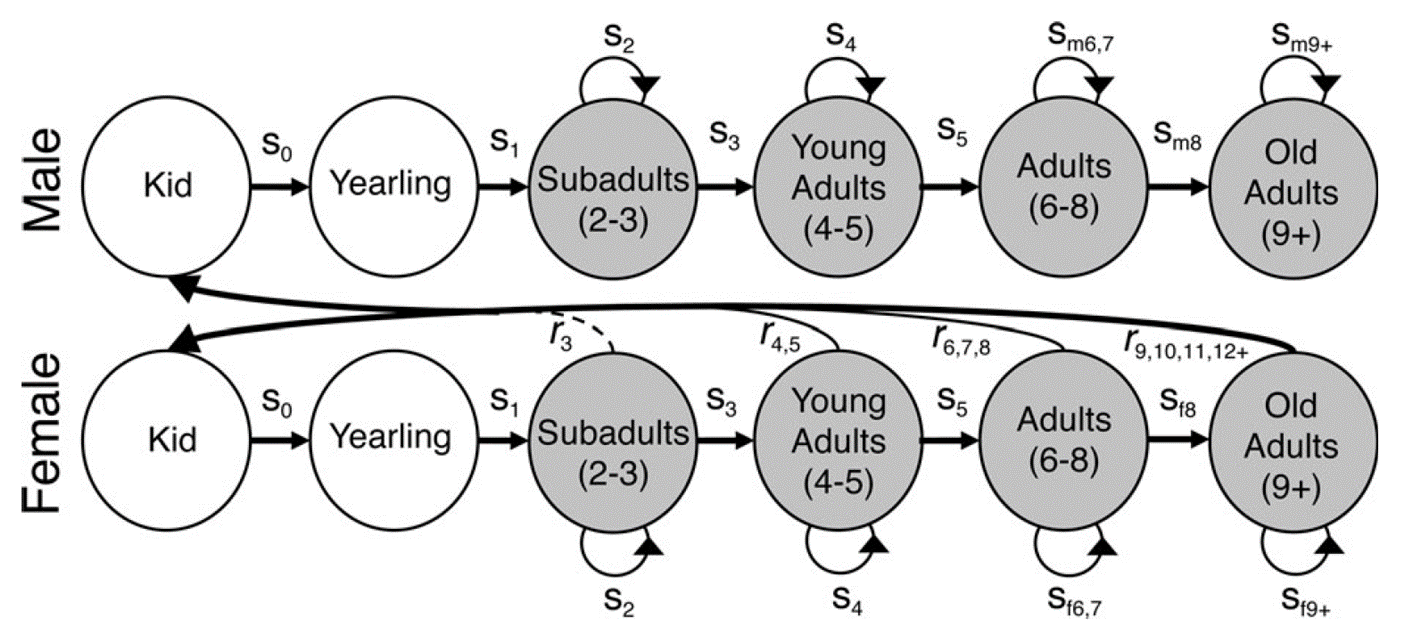


Figure S3. Spatiotemporal variation among populations in avalanche-caused mortality. Proportion of radio-marked mountain goats (n = 421) that died due to avalanches in a given year for each southeastern Alaska study area during 2005 - 2022. Average estimates are depicted by the large colored circles, and small circles represent annual study area estimates. The black vertical line delineates the average across all four study areas and years (adapted from White et al. 2024).


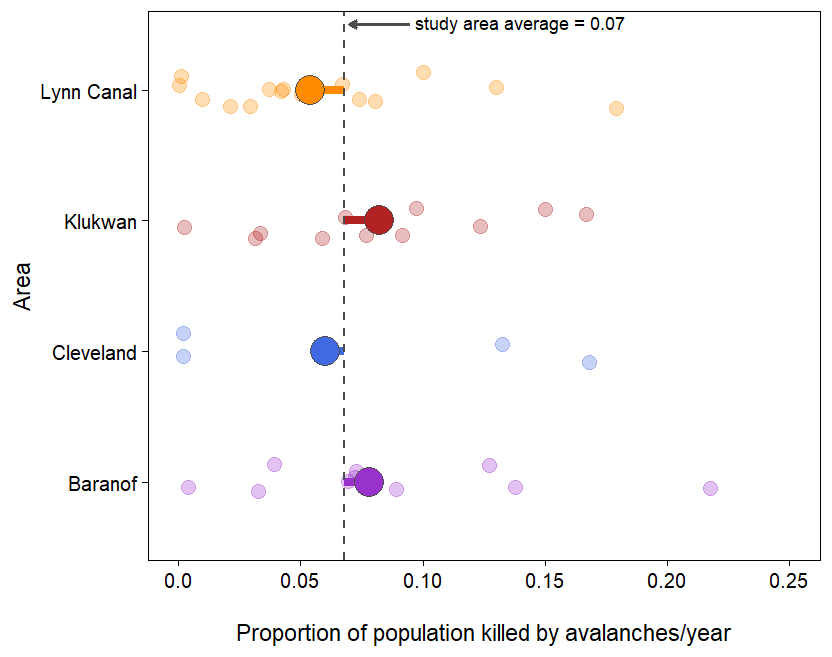


Figure S4. The relationship between the proportion of a sampled mountain goat population dying in avalanches in a given year and total annual survival. Empirical relationships are estimated for each sex-and age category (*sensu* White et al. 2011) and based on radio-marked mountain goats monitored in coastal Alaska during 2005-2021.


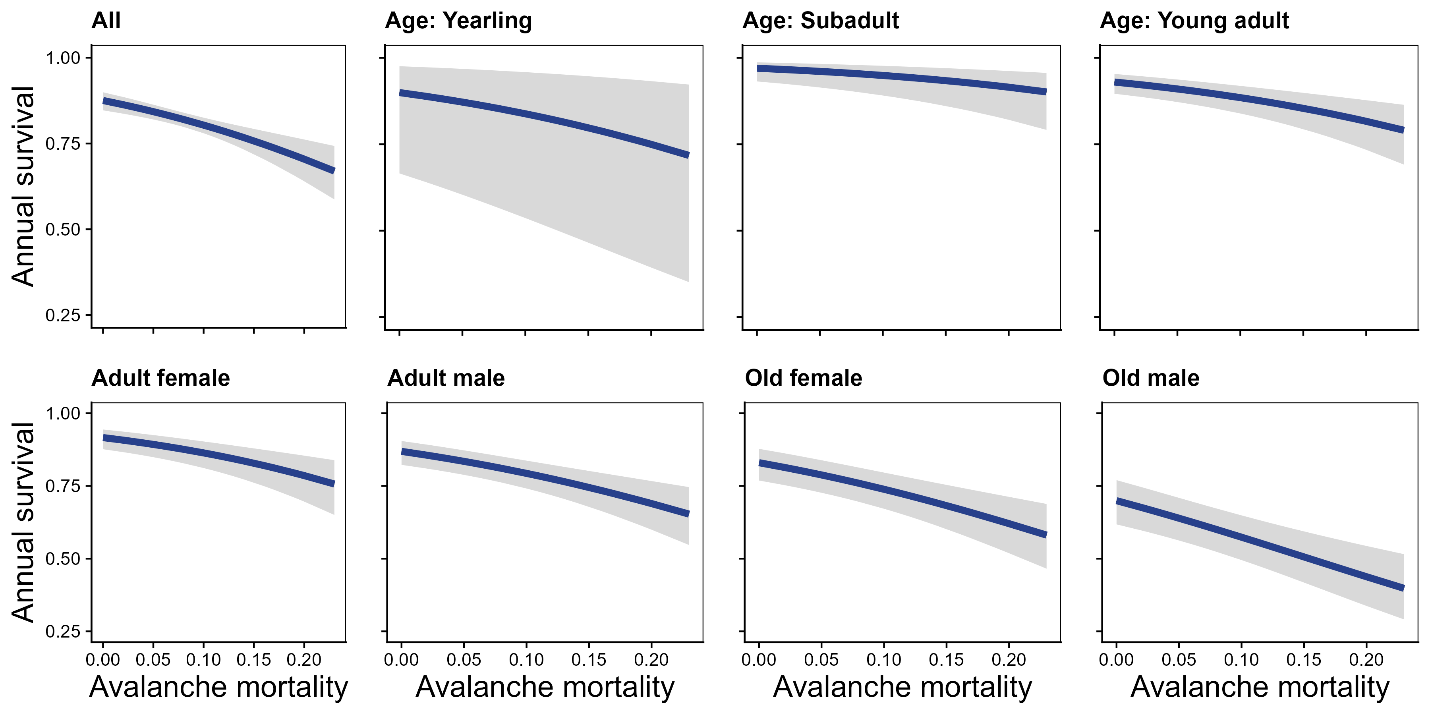


Figure S5. The relationship between proportion of a mountain goat population killed by avalanches across time for four study areas in coastal Alaska during 2005 – 2021. The grey dashed line delineates the threshold level of avalanche mortality (0.088) above which populations are modeled to decline (λ < 1.0 for 50% of simulations or more); below the threshold populations are expected to increase (λ > 1.0).


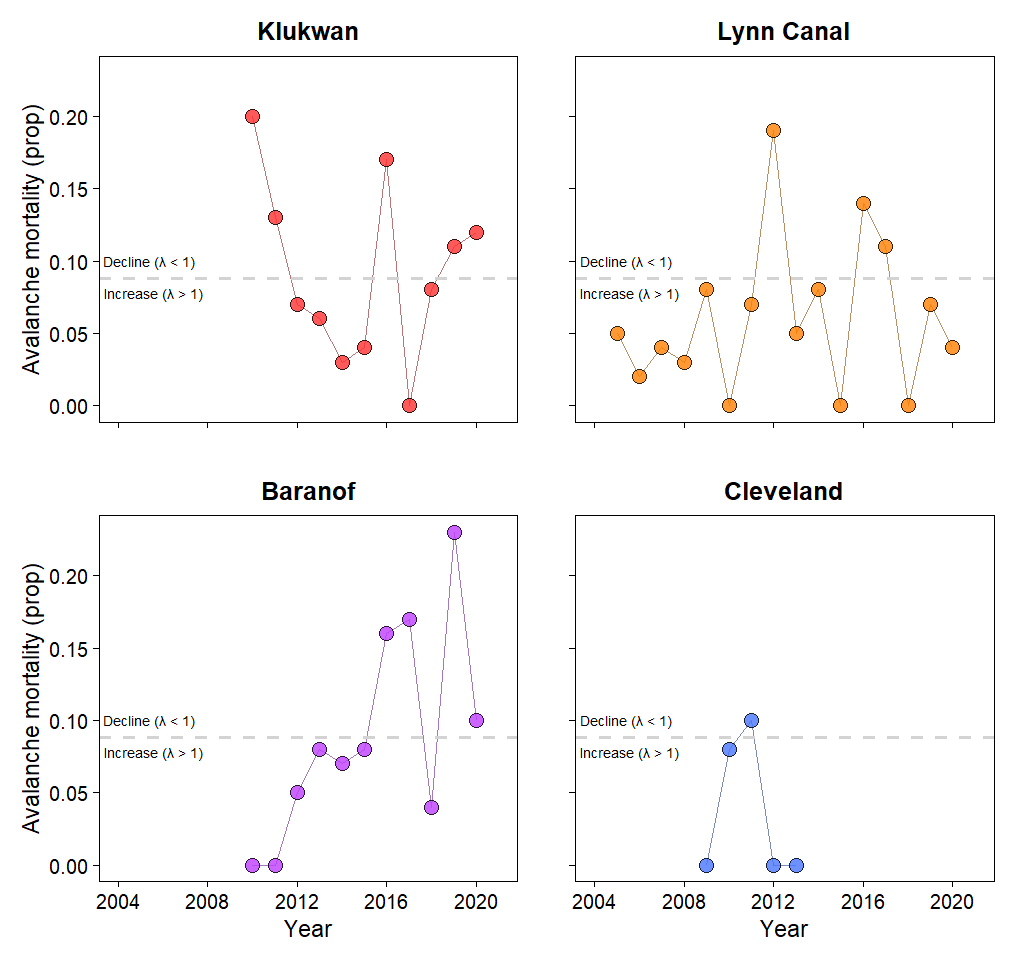


Figure S6. Carnivore scavenging of mountain goat carcasses. (a) Summary of carnivore scavenging activity observed during investigation of radio-collared mountain goat mortalities during 2005 – 2022 in coastal Alaska (n = 148). All species of large- and meso-carnivores that occur in the study area were documented scavenging on mountain goat carcasses. Other large mammalian prey available include: moose (Klukwan, Lynn Canal) and Sitka black-tailed deer (Baranof, Cleveland Peninsula). (b) Wolverine scavenging on a mountain goat carcass in high elevation alpine habitat, Lynn Canal, Alaska. (c) Brown bear (*Ursus arctos*) excavating a mountain goat carcass from an avalanche chute, while a black bear (*Ursus americanus*) observes from distance, Klukwan, Alaska. (Photo credit: K. White).


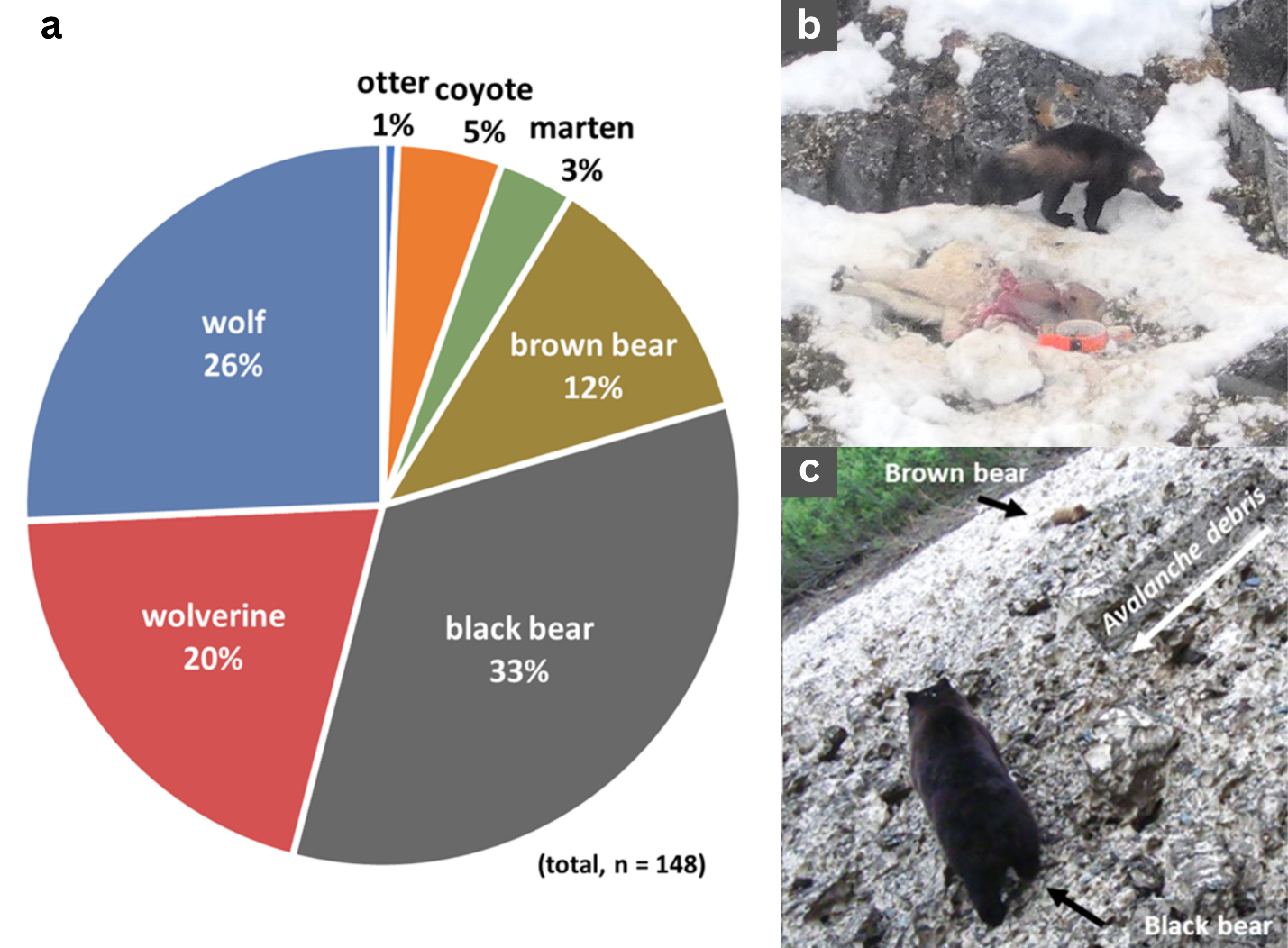

Supplement: Supplementary file 1 — Figure S1: Map depicting mountain goat distribution in Alaska, USA and northwestern Canada. Figure S2: Mountain goat life cycle graph describing the dual‐sex, post‐breeding, age‐structured population model (adapted from White et al. 2018). Figure S3: Spatiotemporal variation among populations in avalanche‐caused mortality. Figure S4: The relationship between the proportion of a sampled mountain goat population dying in avalanches in a given year and total annual survival. Figure S5: The relationship between proportion of a mountain goat population killed by avalanches across time for four study areas in coastal Alaska during 2005–2021. Figure S6: Carnivore scavenging of mountain goat carcasses. [file GCB-31-e70415-s001.docx]
